# Beyond Infection: The Interplay of Salivary Human Herpesvirus 6, Stress, and Host Factors in Major Depressive Disorder

**DOI:** 10.3390/v18060665

**Published:** 2026-06-12

**Authors:** Sunisa Srabuakam, Pitsupha Paladech, Sutida Pongpakdeesakul, Sureewan Duangjit, Sureewan Bumrungthai

**Affiliations:** 1 Faculty of Pharmaceutical Sciences, Ubon Ratchathani University, Ubon Ratchathani 34190, Thailand; 2 Division of Pharmaceutical Chemistry and Technology, Faculty of Pharmaceutical Sciences, Ubon Ratchathani University, Ubon Ratchathani 34190, Thailand; sureewan.d@ubu.ac.th; 3 Division of Biopharmacy, Faculty of Pharmaceutical Sciences, Ubon Ratchathani University, Ubon Ratchathani 34190, Thailand

**Keywords:** HHV-6, major depressive disorder, stress, interaction analysis, Thailand

## Abstract

Human Herpesvirus 6 (HHV-6) is a neurotropic virus associated with lifelong latency and stress-induced reactivation. Its role in major depressive disorder (MDD) remains unclear. This study investigated the association between HHV-6 infection and MDD and evaluated interaction effects with psychosocial and clinical factors. A cross-sectional study was conducted among 2403 university students in Thailand, including 52 participants with physician-diagnosed MDD and 2351 healthy controls. HHV-6 DNA was detected by quantitative polymerase chain reaction (qPCR) using saliva. Logistic regression and interaction analyses were performed. HHV-6 DNA was detected in 50.7% of participants. HHV-6 infection was not significantly associated with MDD (OR = 1.335, 95% CI: 0.766–2.328, *p* = 0.309). Multivariable analysis identified congenital disease, high-fat food consumption, stress, and depressive symptoms as independent predictors of MDD. Significant interaction effects were observed between HHV-6 and several factors. HHV-6 was not independently associated with MDD; however, exploratory interaction analyses identified potential relationships with selected psychosocial and host-related factors that require further validation.

## 1. Introduction

Major depressive disorder (MDD) is a common and recurrent psychiatric disorder characterized by persistent depressed mood, loss of interest or pleasure, cognitive dysfunction, and somatic symptoms that impair daily functioning and quality of life [1,2]. According to the *Diagnostic and Statistical Manual of Mental Disorders, Fifth Edition* (DSM-5), MDD is diagnosed based on depressive symptoms affecting emotional, behavioral, cognitive, and physical domains [1]. Despite advances in pharmacological and psychological treatments, MDD remains a major public health challenge because of its high prevalence, recurrent nature, and long-term impact on morbidity and suicide risk [3,4].

Globally, depression affects approximately 332 million individuals, with an estimated prevalence of 4% in the general population [5]. The condition is more prevalent among women and remains one of the leading causes of disability worldwide [5,6]. In 2023, approximately 766,000 deaths were attributed to suicide, highlighting the substantial burden associated with depressive disorders, particularly among adolescents and young adults [6]. University students represent a vulnerable population because they are frequently exposed to academic demands, social adaptation, financial pressure, and lifestyle changes that may increase psychological distress.

Previous studies have reported a high prevalence of depression and stress among university students worldwide. In Canada, the one-year prevalence of MDD among first-year university students was estimated at approximately 7% in men and 14% in women [7]. Similar trends have been observed globally, with high rates of stress and depression reported among university students in several countries, including Thailand [8,9,10]. In Thailand, depression prevalence among university students ranges from 7.0% to 39.1%, whereas stress prevalence ranges from 15.4% to 48.8% [11]. Furthermore, a multicenter study involving students from 33 Thai universities reported that most students experienced mild to moderate depressive symptoms [12]. These findings emphasize the increasing mental health burden among university students and young adults.

The pathogenesis of MDD is complex and multifactorial. Several biological and psychosocial mechanisms have been proposed, including genetic and epigenetic alterations, neurotransmitter dysregulation, neuroinflammation, hypothalamic–pituitary–adrenal (HPA) axis dysfunction, metabolic abnormalities, chronic medical conditions, lifestyle factors, and environmental stressors [13,14,15,16,17]. Among these factors, chronic stress is considered a major contributor because prolonged exposure to stress may alter neurobiological pathways and increase vulnerability to depressive symptoms [18,19]. Previous evidence has shown that stressful life events frequently precede depressive episodes and are associated with greater symptom severity in both clinical and community populations [20,21].

In Thailand, depression and stress screening are commonly performed using the Srithanya Stress Test-5 (ST-5), Depression Questionnaire (Q2), and Patient Health Questionnaire-9 (PHQ-9), which are widely applied in clinical and community settings [11,22,23]. However, these approaches mainly assess psychological symptoms, while objective biological markers for MDD remain limited. Therefore, identifying potential biological contributors may improve understanding of MDD pathogenesis and support future diagnostic development.

Human herpesvirus 6 (HHV-6) is a neurotropic double-stranded DNA virus belonging to the *Herpesviridae* family [17,24,25]. Following primary infection, HHV-6 establishes lifelong latency and may reactivate during immune dysregulation or stressful conditions [26,27,28]. HHV-6 has been detected in saliva, peripheral blood mononuclear cells, cerebrospinal fluid, and central nervous system tissues [29]. The virus exhibits broad cellular tropism and infects immune cells, astrocytes, and other neural cells involved in central nervous system function [29,30]. Emerging evidence suggests that HHV-6 may contribute to neuroinflammation and neuropsychiatric disorders. Previous studies have linked HHV-6 infection with neurological abnormalities, astrocyte dysfunction, cognitive impairment, and mood disorders [30,31,32,33]. In addition, HHV-6 reactivation has been associated with stress-related conditions and inflammatory pathways implicated in depression [26,27,28]. Given its neurotropic properties, lifelong persistence, and stress-induced reactivation, HHV-6 has emerged as a potential biological factor involved in MDD pathogenesis. However, evidence regarding the association between HHV-6 infection and MDD remains limited and inconsistent, particularly in Thailand. To date, only one study has examined this association in the Thai population, highlighting a substantial gap in the current literature and the need for further investigation [17].

Therefore, this study investigated the association between salivary HHV-6 infection and MDD among 2403 undergraduate students aged 18–24 years in Northeastern Thailand using saliva-based quantitative polymerase chain reaction (qPCR). In addition, interactions between HHV-6 infection and psychosocial, lifestyle, and clinical factors associated with MDD were evaluated.

## 2. Materials and Methods

### 2.1. Specimens

Saliva was collected from 2403 samples of undergraduate students aged 18–24 years old from Northeast Thailand. All participants completed the ST-5, Q-2, and PHQ-9 questionnaires for assessment of stress and depressive symptoms. Participants in the MDD group had a previous diagnosis of major depressive disorder confirmed by a physician. Demographic and clinical information, including sex, health status, congenital diseases, family relationship history, exercise habits, alcohol consumption, and smoking status, was obtained from all participants. Sample size calculation was performed using the formula $N=Z_{1-\alpha }^{2}P(1-P)/d^{2}$, based on an estimated HHV-6 prevalence of approximately 14% [17], with a 95% confidence level (Z = 1.96) and a precision value (d) of 0.01.

Stress levels were screened using the five-item Srithanya Stress Test (ST-5) questionnaire (score range: 0–15), which categorizes participants into four groups: low stress (no problem, score ≤ 4), moderate stress (might have a problem, score 5–7), moderate stress (problem, score 8–9), and high stress (problem, score 10–15). The ST-5 assesses sleep disturbances (insomnia or hypersomnia), impaired concentration, irritability/restlessness/distress, boredom, and social withdrawal over the preceding 2–4 weeks.

The ST-5 questionnaire is widely used in Thailand to assess stress levels. It was developed by selecting five items from a larger pool following validation in 110 staff members at Srithanya Hospital working in inpatient wards. Internal consistency and Pearson’s correlation coefficients were calculated and compared with the Hospital Anxiety and Depression Scale (HADS). The HADS and self-perceived stress across seven related conditions were used as reference standards for validity. The ST-5 items demonstrated strong correlations with HADS measures. Specifically, ST-5 scores correlated with HADS-Anxiety and HADS-Depression scores (r = 0.58 and 0.59, respectively; *p* < 0.01), while the correlation between HADS-Anxiety and HADS-Depression was r = 0.61 (*p* < 0.01) at ST-5 scores > 7.

The Patient Health Questionnaire (Q)-2 and PHQ-9 were used to screen for depression. Q-2 scores of 1 and 2 were interpreted as no depression and at risk of depression, respectively, over the preceding two weeks. PHQ-9 scores (range 0–27) were interpreted as no depression (0–7), mild depression (8–12), moderate depression (13–17), and major depression (≥18). A PHQ-9 score ≥ 9 was used to classify depression [11,22,23]. These instruments were used for descriptive and covariate analyses.

Inclusion criteria: The study population consisted of community-based volunteers residing in Thailand. Eligible participants were individuals aged 18–24 years with permanent residence in Thailand who were willing to participate in the study. Only participants who were assessed as clinically stable and not classified as medically vulnerable were considered eligible for inclusion. Exclusion criteria: Individuals were excluded if they had significant or unstable comorbid medical conditions, declined venous saliva collection, refused to complete the study questionnaire, or were unable to provide informed consent due to impaired cognitive capacity or any condition limiting independent decision-making.

This study was approved by the Ubon Ratchathani University Research Ethics Committee UBU-REC-69/2567 (2 July 2024–1 July 2025 and 2 July 2025–1 July 2026). All study procedures involving human participants were conducted in accordance with the ethical principles outlined in the Declaration of Helsinki, the Belmont Report, the Council for International Organizations of Medical Sciences (CIOMS) guidelines, and the International Conference on Harmonization Good Clinical Practice (ICH-GCP) guidelines. Written informed consent was obtained from all participants and/or their legal guardians prior to sample collection.

### 2.2. DNA Extraction

A total of 300 µL of saliva was transferred from the collection tube into a microcentrifuge tube. Cell lysis was performed by adding 160 µL of 2.5× lysis buffer, followed by enzymatic digestion using Proteinase K (11 mg/mL; 10 µL) and lysozyme (50 mg/mL; 20 µL). The mixture was vortexed for 3–5 min until the pellet was completely resuspended without visible aggregates. Samples were incubated at 37 °C for 30 min and subsequently at 65 °C for 1 h; the Proteinase K digestion step could be extended to 1 h when required. The samples were then cooled to room temperature for 20 min. Protein precipitation was achieved by adding 400 µL of 5 M potassium acetate, followed by gentle mixing through repeated inversion (~50 times). Samples were kept on ice at 4 °C for 10 min to facilitate protein precipitation and centrifuged at 12,000× *g* at 4 °C for 10 min. Subsequently, 500 µL of the clear supernatant was transferred to a new tube and mixed with an equal volume (500 µL) of isopropanol by inversion (~50 times). The mixture was incubated at 4 °C for 10 min prior to centrifugation at 12,000× *g* at 4 °C for 10 min to pellet the DNA. The DNA pellet was washed with 500 µL of 70% ethanol and centrifuged at 12,000× *g* at 4 °C for 1 min. After removal of the supernatant, the pellet was air-dried for 30–60 min and resuspended in 150 µL of TE buffer. The dissolved DNA was incubated at 65 °C for 15 min and stored at −20 °C until further analysis.

### 2.3. HHV-6 DNA Detection by Quantitative Polymerase Chain Reaction (qPCR)

HHV-6 DNA targeting the *U97* gene was detected by quantitative polymerase chain reaction (qPCR) using the forward primer 5′-GCTAGAACGTATTTGCTGCAGAACG-3′ and reverse primer 5′-ATCCGAAACAACTGTCTGACTGGCA-3′, generating a 259 bp amplicon. An in-house plasmid containing the 259 bp *U97* fragment served as the positive control. All reactions were performed in duplicate. qPCR amplification was carried out using 5× FiREPOL qPCR Mix Plus (Solis BioDyne, Tartu, Estonia) following the protocol described previously [17]. The *GAPDH* gene was used as an internal control, including the forward primer 5′-TCATCAGCAATGCCTCCTGCA-3′ and reverse primer 5′-TGGGTGGCAGTGATGGCA-3′, yielding a 117 bp amplicon.

### 2.4. Statistical Analysis

Statistical analyses were performed using IBM SPSS Statistics version 16 (IBM Corp., Armonk, NY, USA). Continuous variables were expressed as mean ± standard deviation (SD), while categorical variables were presented as frequencies and percentages. Group comparisons were conducted using the independent *t*-test, chi-square test, or Fisher’s exact test, as appropriate. Binary logistic regression analysis was performed to identify factors associated with MDD, and adjusted odds ratios (AORs) with 95% confidence intervals (95% CIs) were calculated. Interaction analyses were conducted by incorporating interaction terms into the logistic regression model to evaluate the combined effects of HHV-6 infection and related variables on MDD. A two-tailed *p* value < 0.05 was considered statistically significant.

## 3. Results

### 3.1. Baseline Characteristics

A total of 2403 participants were included, comprising 52 individuals with MDD and 2351 healthy controls. HHV-6 DNA was detected in 1218 participants (50.7%). No significant association was observed between HHV-6 positivity and MDD status (OR = 1.335, 95% CI: 0.766–2.328, *p* = 0.307). Similarly, HHV-6 detection was not significantly associated with family history, alcohol consumption, smoking, dietary habits, exercise frequency or most environmental factors (Table 1 and Table 2 and Supplementary Table S1).

The mean age of participants with MDD was slightly higher than that of non-depressed participants (20.48 ± 1.16 vs. 20.16 ± 1.24 years); however, the difference was not statistically significant (*p* = 0.053) (Table 3).

Participants with MDD had significantly lower height compared with healthy participants (161.21 ± 6.86 cm vs. 164.62 ± 8.57 cm, *p* = 0.004). In contrast, no significant differences were observed for body weight (59.77 ± 17.46 kg vs. 61.09 ± 16.08 kg, *p* = 0.592) or BMI (22.93 ± 6.28 vs. 22.42 ± 5.07 kg/m^2^, *p* = 0.570) (Table 3).

Psychological assessments demonstrated markedly higher scores among participants with MDD. The mean ST-5 stress score was significantly elevated in the MDD group compared with non-depressed participants (8.58 ± 3.21 vs. 5.41 ± 2.42, *p* < 0.001), with a mean difference 95% CI of 2.49–3.84. Similarly, PHQ-9 scores were substantially higher in participants with MDD (10.90 ± 5.90 vs. 5.39 ± 3.63, *p* < 0.001), with a mean difference 95% CI of 4.49–6.58 (Table 2).

Poor family relationships were strongly associated with MDD (*p* < 0.001). Participants with congenital disease had approximately six-fold higher odds of MDD compared with those without congenital disease (OR = 5.935, 95% CI: 3.338–10.551, *p* < 0.001). High-fat food consumption was also significantly associated with increased MDD risk (OR = 2.999, 95% CI: 1.251–7.189, *p* = 0.010). In addition, PHQ-9 severity and ST-5 stress scores showed strong associations with MDD (*p* < 0.001 for both).

Overall, participants with MDD exhibited significantly greater psychological stress and depressive symptom severity, whereas anthropometric measures such as weight and BMI did not differ significantly between groups.

Regarding HHV-6 infection, age was significantly associated with HHV-6 detection (*p* = 0.025), indicating variation in infection prevalence across age groups. Faculty affiliation was also significantly associated with HHV-6 positivity (*p* < 0.001), with higher prevalence observed among science-related faculties. Participants from science-stream programs showed significantly higher HHV-6 positivity than those from arts-stream programs (OR = 1.989, 95% CI: 1.667–2.373, *p* < 0.001). Additionally, participants who used tap water for tooth brushing showed a lower likelihood of HHV-6 positivity (OR = 0.697, 95% CI: 0.504–0.965, *p* = 0.029) (Table 2).

Overall, HHV-6 infection was not associated with MDD in this cohort. Instead, MDD was more strongly linked to psychosocial factors, congenital disease, dietary behavior, and psychological stress indicators.

### 3.2. Univariate Analysis

Logistic regression showed no association between HHV-6 and MDD. Female sex, congenital disease, high-fat diet, poor family relationships, and psychological stress were significant risk factors, while science-stream enrollment and good family relationships were protective. Depressive severity (Q2/PHQ-9) strongly increased MDD odds. Other variables were not significant (Table 4).

Overall, MDD appeared to be predominantly associated with psychosocial, behavioral, and health-related factors rather than HHV-6 infection.

### 3.3. Multivariable Analysis

Multivariable logistic regression analysis identified several independent factors associated with MDD. Height remained significantly associated with MDD after adjustment for confounding factors (OR = 1.048, 95% CI: 1.005–1.093, *p* = 0.030). Participants with congenital disease had approximately four-fold higher odds of MDD compared with those without underlying diseases (OR = 3.902, 95% CI: 2.012–7.567, *p* < 0.001). Dietary behavior also contributed to MDD risk. Participants with high-fat food consumption had significantly increased odds of depression, with approximately 4.5-fold higher risk compared with the reference group (OR = 4.487, 95% CI: 1.669–12.063, *p* = 0.003). Psychological stress remained a strong independent predictor of MDD (Table 5).

### 3.4. Interaction Analysis

Interaction analysis showed that HHV-6 infection significantly interacted with multiple demographic, clinical, lifestyle, and psychological factors. Significant interactions were observed with sex (OR = 2.412, *p* = 0.006), academic factors including faculty and science–arts stream (OR = 0.513–0.931, *p* ≤ 0.006), and family relationships, where better relationships were protective (OR = 0.777, *p* = 0.037) and poor relationships increased risk (OR = 2.873, *p* = 0.023). Clinical and lifestyle interactions included body weight, BMI, congenital disease (OR = 2.753, *p* < 0.001), high-fat diet (OR = 2.671, *p* = 0.006), and fruit/vegetable intake (OR = 0.405–0.709, *p* ≤ 0.013). Psychological factors also showed significant interactions, including chronic stress, ST-5, and PHQ-9 scores, with several higher PHQ-9 categories showing elevated risk (OR = 2.454, *p* = 0.001). Higher-order models combining sex, age, congenital disease, stress, diet, and environmental factors were also significant (*p* ≤ 0.010), particularly stress- and family-related combinations (*p* < 0.001) (Table 6).

Several interaction terms were significant before correction for multiple comparisons; however, only a subset remained significant after Bonferroni adjustment (α = 0.002). Therefore, these findings should be considered exploratory and interpreted with caution.

Overall, although HHV-6 was not directly associated with MDD, it may modulate risk through complex interaction effects across psychosocial, clinical, and lifestyle domains.

## 4. Discussion

The absence of a direct association between salivary HHV-6 and MDD in the present study does not necessarily exclude a potential biological role of HHV-6 in depression. Although HHV-6 infection alone was not significantly associated with MDD (*p* = 0.309). After correction for multiple comparisons using the Bonferroni method, significant interaction effects remained for stress status and congenital disease. These findings suggest that the potential influence of HHV-6 on MDD susceptibility may vary according to specific host-related factors. However, given the exploratory nature of the interaction analyses and the limited number of MDD cases, these results should be interpreted with caution and require confirmation in larger prospective studies.

Psychological stress has been consistently recognized as a major contributor to MDD pathogenesis and has been implicated in the reactivation of latent herpesviruses, including HHV-6. HHV-6 establishes lifelong latency following primary infection and may reactivate during periods of stress, immune dysregulation, or illness. Reactivation has been associated with inflammatory responses, neuroimmune activation, and neurological manifestations in both experimental and clinical studies [34,35]. Therefore, stress-induced HHV-6 reactivation has been proposed as a potential mechanism linking viral persistence to neuropsychiatric outcomes. However, because the present study assessed HHV-6 DNA in saliva, the findings cannot distinguish latent viral carriage from active reactivation and do not provide evidence regarding viral activity within the central nervous system [28,35,36,37,38,39].

Several biological mechanisms have been proposed in the pathogenesis of MDD, including dysregulation of the HPA axis, monoaminergic dysfunction, inflammatory responses, genetic and epigenetic alterations, structural and functional brain changes, and psychosocial factors [40]. Among these, chronic or recurrent psychological stress is considered a major precipitating factor for depressive episodes [41]. Since stress may also promote herpesvirus reactivation, interactions between stress exposure and latent viral infection may represent an additional pathway influencing susceptibility to MDD. Nevertheless, the present study was not designed to evaluate viral reactivation directly, and no conclusions regarding the biological activity of HHV-6 can be drawn from salivary DNA detection alone.

HHV-6 has attracted interest as a potential contributor to neuropsychiatric disorders because of its neurotropic properties and lifelong persistence. The virus preferentially infects CD4^+^ T lymphocytes but can also infect endothelial, epithelial, and central nervous system (CNS) cells, allowing persistence within neural tissues [29]. Previous studies have reported increased detection of HHV-6 DNA and viral proteins in cerebellar tissues of patients with MDD and bipolar disorder, particularly within Purkinje cells, suggesting possible involvement in neuronal dysfunction [17,32,42].

Experimental evidence further suggests that HHV-6 may influence neuroimmune pathways through induction of inflammatory mediators and oxidative stress responses, including cytokines such as TNF-α and IL-1. HHV-6 infection has also been shown to alter expression of genes associated with neurological function and CNS disorders [30]. In addition, latent HHV-6B infection in olfactory astrocytes has been associated with SITH-1 expression, apoptosis, and depression-like phenotypes in experimental models. However, these findings remain largely mechanistic and do not establish a direct causal relationship with MDD in humans. Moreover, the present study does not provide evidence supporting viral reactivation, systemic dissemination, neuroinflammation, or CNS involvement. Therefore, the biological mechanisms discussed above are derived from previous experimental and clinical studies and should not be interpreted as mechanisms demonstrated by the current findings.

Importantly, not all individuals infected with HHV-6 develop depression, and most infected individuals remain asymptomatic. Together with our findings showing no independent association between HHV-6 and MDD, these observations support the hypothesis that HHV-6 may act as a cofactor rather than an independent etiological agent. Its potential effects may become evident only under specific host, psychosocial, or environmental conditions, particularly in the presence of stress-related viral reactivation and immune dysregulation.

This study has several limitations. First, the cross-sectional design precludes causal inference between HHV-6 infection and MDD. Second, salivary HHV-6 DNA detection could not distinguish latent infection from active viral reactivation. Third, although the overall sample size was relatively large, the small number of MDD cases (*n* = 52) and the marked imbalance between cases and controls may have substantially reduced statistical power, thereby limiting our ability to detect modest associations between HHV-6 infection and MDD. This imbalance may also have increased the susceptibility of the regression analyses to unstable estimates and the influence of outliers, particularly for less common factors. Therefore, the non-significant association observed between HHV-6 infection and MDD should be interpreted cautiously, as a lack of statistical significance does not necessarily indicate the absence of a true association. MDD status was determined based on self-reported physician diagnosis rather than a structured psychiatric interview. As a result, some participants in the control group may have experienced depressive symptoms or undiagnosed depressive disorders, which could have led to misclassification and biased the observed associations toward the null. Finally, multiple interaction analyses were performed, potentially increasing the risk of false-positive findings; therefore, replication in larger prospective studies is warranted. Future studies using peripheral blood and markers of viral reactivation may better distinguish latent infection from active HHV-6 reactivation.

## 5. Conclusions

Overall, 2403 participants were included, with an HHV-6 prevalence of 50.7%. Salivary HHV-6 DNA was not independently associated with MDD. Female sex, poor family relationships, congenital disease, high-fat food consumption, stress, and depressive symptom severity were associated with MDD. While exploratory interaction analyses suggested potential relationships between HHV-6 and selected host factors, these findings require cautious interpretation and independent validation. Given the limitations of salivary HHV-6 detection, the present study does not support a direct association between HHV-6 infection and MDD.

## Figures and Tables

**Table 1:** Characteristics of Study Participants (N = 2403).

Characteristic	Category	Total *n* (%)
Age (years)	18	170 (7.1)
	19	600 (25.0)
	20	727 (30.3)
	21	580 (24.1)
	22	239 (9.9)
	23	69 (2.9)
	24	18 (0.7)
Year of study	First year	647 (26.9)
	Second year	830 (34.5)
	Third year	638 (26.6)
	Fourth year	242 (10.1)
	Fifth year	46 (1.9)
Academic stream	Science	1661 (69.1)
	Arts	742 (30.9)
Family relationship	Very good	1395 (58.1)
	Good	663 (27.6)
BMI category	Underweight	454 (18.9)
	Normal	1393 (58.0)
	Overweigh	340 (14.1)
	Obesity class I	140 (5.8)
	Obesity class II	76 (3.2)
Congenital disease	Yes	244 (10.2)
	No	2159 (89.8)
Alcohol consumption	Yes	1958 (81.5)
	No	445 (18.5)
Smoking	Yes	327 (13.6)
	No	2076 (86.4)
PHQ-9 category	No depression	1440 (59.9)
	Mild depression	856 (35.6)
	Moderate depression	94 (3.9)
	Severe depression	13 (0.5)
ST-5 category	No stress	735 (30.6)
	Mild stress	1247 (51.9)
	Moderate stress	262 (10.9)
	Severe stress	159 (6.6)
MDD status	MDD	52 (2.2)
	Healthy controls	2351 (97.8)
HHV-6 status	Positive	1218 (50.7)
	Negative	1185 (49.3)

**Table 2:** Baseline characteristics and univariate analysis of factors associated with MDD and HHV-6 infection.

Factor	Disease			Odds Ratio (95% CI)	*p*-Value	HHV-6			Odds Ratio (95% CI)	*p*-Value
	*n*	MDD	Healthy			*n*	Positive	Negative		
		(N = 52)	(N = 2351)				(N = 1218)	(N = 1185)		
HHV-6										
Positive	1218	30 (57.7%)	1188 (50.5%)	1.335 (0.766–2.328)	0.307	-	-	-	-	-
Negative	1185	22 (42.3%)	1163 (49.5%)	Ref						
Disease										
MDD	-	-	-	-	-	52	30 (57.7%)	22 (42.3%)	1.335 (0.766–2.328)	0.307
Healthy						2351	1188 (50.5%)	1163 (49.5%)	Ref	
Age										
18 years	170	2 (3.8%)	168 (7.1%)	N/A	0.133	170	101 (8.3%)	69 (5.8%)	N/A	0.025
19 years	600	6 (11.5%)	594 (25.3%)			600	293 (24.1%)	307 (25.9%)		
20 years	727	22 (42.3%)	705 (30.0%)			727	382 (31.4%)	345 (29.1%)		
21 years	580	12 (23.1%)	568 (24.2%)			580	287 (23.6%)	293 (24.7%)		
22 years	239	7 (13.5%)	232 (9.9%)			239	121 (9.9%)	118 (10%)		
23 years	69	3 (5.8%)	66 (2.8%)			69	24 (2.0%)	45 (3.8%)		
24 years	18	0 (0%)	18 (0.8%)			18	10 (0.8%)	8 (0.7%)		
Year of study										
First year	647	9 (17.3%)	638 (27.1%)	N/A	0.326	647	347 (28.5%)	300 (25.3%)	N/A	0.213
Second year	830	22 (42.3%)	808 (34.4%)			830	424 (34.8%)	406 (34.3%)		
Third year	638	12 (23.1%)	626 (26.6%)			638	306 (25.1%)	332 (28.0%)		
Fourth year	242	7 (13.5%)	235 (10.0%)			242	122 (10.0%)	120 (10.1%)		
Fifth year	46	2 (3.8%)	44 (1.9%)			46	19 (1.6%)	27 (2.3%)		
Faculty										
Agriculture	304	3 (5.8%)	301 (12.8%)	N/A	0.097	304	196 (16.1%)	108 (9.1%)	N/A	0.000
Law	306	8 (15.4%)	298 (12.7%)			306	122 (10.0%)	184 (15.5%)		
Business	132	2 (3.8%)	130 (5.5.%)			132	55 (4.5%)	77 (6.5%)		
Nursing	150	2 (3.8%)	148 (6.3%)			150	57 (4.7%)	93 (7.8%)		
Pharmacy	342	6 (11.5%)	336 (14.3%)			342	229 (18.8%)	113 (9.5%)		
Politics	216	8 (15.4%)	208 (8.8%)			216	73 (6.0%)	143 (12.1%)		
Science	121	2 (3.8%)	119 (5.1%)			121	78 (6.4%)	43 (3.6%)		
Engineering	469	6 (11.5%)	463 (19.7%)			469	263 (21.6%)	206 (17.4%)		
Arts	44	3 (5.8%)	41 (1.7%)			44	17 (1.4%)	27 (2.3%)		
Education	44	2 (3.8%)	42 (1.8%)			44	22 (1.8%)	22 (1.9%)		
Public Health	275	10 (19.2%)	265 (11.3%)			275	106 (8.7%)	169 (14.3%)		
Science and Arts Faculty Group										
Science stream	1661	29 (55.8%)	1632 (69.4%)	0.555 (0.319–0.967)	0.035	1661	929 (76.3%)	732 (61.8%)	1.989 (1.667–2.373)	0.000
Arts stream	742	23 (44.2%)	719 (30.6%)	Ref		742	283 (23.7%)	453 (38.2%)	Ref	
Family relationship										
Very good	1395	22 (42.3%)	1373 (58.4%)	N/A	0.000	1395	719 (59.0%)	676 (57.0%)	N/A	0.431
Good	663	10 (19.2%)	653 (27.8%)			663	334 (27.4%)	329 (27.8%)		
Normal	323	17 (32.7%)	306 (13.0%)			323	152 (12.5%)	171 (14.4%)		
Quarreling	22	3 (5.8%)	19 (0.8%)			22	13 (1.1%)	9 (0.8%)		
BMI										
Below normal	454	12 (23.1%)	442 (18.8%)	N/A	0.276	454	247 (20.3%)	207 (17.5%)	N/A	0.067
Normal	1393	29 (55.8%)	1364 (58.0%)			1393	715 (58.7%)	678 (57.2%)		
Overweight	340	5 (9.6%)	335 (14.2%)			340	151 (12.4%)	189 (15.9%)		
Obesity class I	140	2 (3.8%)	138 (5.9%)			140	70 (5.7%)	70 (5.9%)		
Obesity class II	76	4 (7.7%)	72 (3.1%)			76	35 (2.9%)	41 (3.5%)		
Congenital disease										
Yes	244	20 (38.5%)	224 (9.5%)	5.935 (3.338–10.551)	0.000	244	134 (11.0%)	110 (9.3%)	1.208 (0.926–1.576)	0.163
No	2159	32 (61.5%)	2127 (90.5%)	Ref		2159	1084 (89.0%)	1075 (90.7%)	Ref	
Family history										
Yes	777	21 (40.4%)	756 (32.2%)	1.429 (0.816–2.504)	0.210	777	395 (32.4%)	382 (32.2%)	1.009 (0.850–1.197)	0.919
No	1626	31 (59.6%)	1595 (67.8%)	Ref		1626	823 (67.6%)	803 (67.8%)	Ref	
Alcohol consumption										
Yes	1958	45 (86.5%)	1913 (81.4%)	1.472 (0.659–3.286)	0.343	1958	980 (80.5%)	978 (82.5%)	0.872 (0.709–1.071)	0.191
No	445	7 (13.5%)	438 (18.6%)	Ref		445	238 (19.5%)	207 (17.5%)	Ref	
Smoking status										
Yes	327	10 (19.2%)	317 (13.5%)	1.528 (0.759–3.076)	0.232	327	170 (14.0%)	157 (13.2%)	1.062 (0.841–1.341)	0.613
No	2076	42 (80.8%)	2034 (86.5%)	Ref		2076	1048 (86.0%)	1028 (86.8%)	Ref	
Secondhand smoke										
Yes	950	23 (44.2%)	927 (39.4%)	1.218 (0.700–2.119)	0.484	950	466 (38.3%)	484 (40.8%)	0.898 (0.762–1.057)	0.195
No	1453	29 (55.8%)	1424 (60.6%)	Ref		1453	752 (61.7%)	701 (59.2%)	Ref	
Nutritionally adequate diet										
No	1068	23 (44.2%)	1045 (44.4%)	0.991 (0.570–1.724)	0.975	1068	539 (44.3%)	529 (44.6%)	0.984 (0.838–1.156)	0.848
Yes	1335	29 (55.8%)	1306 (55.6%)	Ref		1335	679 (55.7%)	656 (55.4%)	Ref	
Exercise > 30 min										
No	777	23 (44.2%)	754 (32.1%)	N/A	0.243	777	372 (30.5%)	405 (34.2%)	N/A	0.159
1–2 times/week	1140	22 (42.3%)	1118 (47.6%)			1140	582 (47.8%)	558 (47.1%)		
3–4 times/week	358	6 (11.5%)	352 (15.0%)			358	193 (15.8%)	165 (13.9%)		
5–7 times/week	128	1 (1.9%)	127 (5.4%)			128	71 (5.8%)	57 (4.8%)		
Fresh fruit consumption										
No	137	5 (9.6%)	132 (5.6%)	1.788 (0.700–4.571)	0.218	137	60 (4.9%)	77 (6.5%)	0.746 (0.527–1.055)	0.097
Yes	2266	47 (90.4%)	2219 (94.4%)	Ref		2266	1158(95.1%)	1108(93.5%)	Ref	
High-fat food consumption										
No	104	6 (11.5%)	98 (4.2%)	2.999 (1.251–7.189)	0.010	104	55 (4.5%)	49 (4.1%)	1.096 (0.740–1.625)	0.647
Yes	2299	46 (88.5%)	2253 (95.8%)	Ref		2299	1163 (95.5%)	1136 (95.9%)	Ref	
Fermented food consumption										
No	794	19 (36.5%)	775 (33.0%)	1.171 (0.662–2.072)	0.588	794	418 (34.3%)	376 (31.7%)	1.124 (0.948–1.333)	0.177
Yes	1609	33 (63.5%)	1576 (67.0%)	Ref		1609	800 (65.7%)	809 (68.3%)	Ref	
Household water consumption										
Unclean	721	18 (34.6%)	703 (29.9%)	1.241 (0.696–2.212)	0.463	721	367 (30.1%)	354 (29.9%)	1.012 (0.850–1.205)	0.890
Clean	1682	34 (65.4%)	1648 (70.1%)	Ref		1682	851 (69.9%)	831 (70.1%)	Ref	
Boiled or filtered water consumption										
No	803	20 (38.5%)	783 (33.3%)	1.252 (0.711–2.203)	0.436	803	393 (32.3%)	410 (34.6%)	0.900 (0.760–1.067)	0.225
Yes	1600	32 (61.5%)	1568 (66.7%)	Ref		1600	825 (67.7%)	775 (65.4%)	Ref	
Brushing teeth with tap water										
Yes	2242	46 (88.5%)	2196 (93.4%)	0.541 (0.228–1.287)	0.158	2242	1123 (92.2%)	1119 (94.4%)	0.697 (0.504–0.965)	0.029
No	161	6 (11.5%)	155 (6.6%)	Ref		161	95 (7.8%)	66 (5.6%)	Ref	
PHQ-9										
No depression	1440	12 (23.1%)	1428 (60.7%)	N/A	0.000	1440	732 (60.1%)	708 (59.7%)	N/A	0.888
Mild	856	17 (32.7%)	839 (35.7%)			856	435 (35.7%)	421 (35.5%)		
Moderate	94	20 (38.5%)	74 (3.1%)			94	44 (3.6%)	50 (4.2%)		
Severe	13	3 (5.8%)	10 (0.4%)			13	7 (0.6%)	6 (0.5%)		
ST-5										
No stress	735	5 (9.6%)	730 (31.1%)	N/A	0.000	735	385 (31.6%)	350 (29.5%)	N/A	0.156
Mild stress	1247	15 (28.8%)	1232 (52.4%)			1247	635 (52.1%)	612 (51.6%)		
Moderate stress	262	11 (21.2%)	251 (10.7%)			262	116 (9.5%)	146 (12.3%)		
Severe stress	159	21 (40.4%)	138 (5.9%)			159	82 (6.7%)	77 (6.5%)		

**Table 3:** Comparison of continuous demographic, anthropometric, and psychological variables between depressed and non-depressed participants.

Factor	MDD	*n*	Mean	Std. Deviation	Std. Error Mean	*p*-Value	95% Confidence Interval of the Difference (CI)	
							Lower	Upper
Age	Depressed	52	20.4808	1.16300	0.16128	0.053	−0.00487	0.64995
	Not depressed	2351	20.1582	1.23515	0.02547			
Weight	Depressed	52	59.7692	17.45989	2.42125	0.592	−6.22068	3.58314
	Not depressed	2351	61.0880	16.08101	0.33166			
BMI (Body Mass Index)	Depressed	52	22.9255	6.27798	0.87060	0.570	−1.25794	2.26041
	Not depressed	2351	22.4243	5.07449	0.10466			
ST-5 score (0–15) *	Depressed	52	8.5769	3.20750	0.44480	0.000	2.49110	3.83501
	Not depressed	2351	5.4139	2.42490	0.05001			
PHQ-9 score (0–27) **	Depressed	52	10.9038	5.89869	0.81800	0.000	4.48570	6.57848
	Not depressed	2351	5.3718	3.74774	0.07729			

* The ST-5 questionnaire was used to screen for stress conditions across four categories based on a score of 0–5: low stress levels (no problem, score ≤ 4), moderate stress (might have a problem, 5–7), moderate stress (problem, 8–9), and high stress levels (problem, 10–15). The questionnaire assessed symptoms experienced including sleep problems (insomnia and excessive sleeping), low concentration, feeling irritated/restless/distraught, feeling bored, and social withdrawal over the previous 2–4 weeks. ** Depression screening was performed using Q2 and PHQ-9 tools. Q2 classified response into no depression (score 1) or at risk for depression (score 1) based on symptoms over the past 2 weeks; 2) PHQ-9, scored 0–27, were interpreted as no depression as none (0–7), mild (8–12), moderate (13–17), or major depression (≥18). PHQ-9 score ≥ 9 was classified as depression.

**Table 4:** Univariate logistic regression analysis of factors associated with MDD, including HHV-6 infection.

Factor	B	S.E.	Wald	df	*p*-Value	OR	95% CI	
							Lower	Upper
HHV-6	0.289	0.284	1.037	1	0.309	1.335	0.766	2.328
Sex	1.221	0.436	7.832	1	0.005	3.391	1.442	7.976
Age	−0.203	0.109	3.459	1	0.063	0.816	0.659	1.011
Year of study	−0.177	0.133	1.775	1	0.183	0.838	0.646	1.087
Faculty	−0.060	0.033	3.268	1	0.071	0.941	0.882	1.005
Science–Arts stream group	−0.588	0.283	4.322	1	0.038	0.555	0.319	0.967
Family relationship	2.017	0.638	9.999	1	0.002	7.515	2.153	26.230
Weight	0.005	0.009	0.341	1	0.559	1.005	0.987	1.024
BMI	−0.018	0.026	0.491	1	0.484	0.982	0.934	1.033
Congenital disease	1.781	0.294	36.797	1	0.000	5.935	3.338	10.551
Nutritionally adequate diet	−0.009	0.282	0.001	1	0.975	0.991	0.570	1.724
Exercise > 30 min	0.368	0.190	3.764	1	0.052	1.445	0.996	2.096
Fresh fruit consumption	−0.210	0.188	1.244	1	0.265	0.810	0.560	1.172
Vegetable consumption	0.022	0.169	0.016	1	0.899	1.022	0.733	1.424
High-fat diet consumption	1.098	0.446	6.059	1	0.014	2.999	1.251	7.189
Fermented food consumption	0.094	0.217	0.187	1	0.665	1.098	0.718	1.680
Household water	−0.139	0.199	0.486	1	0.486	0.870	0.589	1.285
Boiled or filtered water consumption	0.224	0.288	0.606	1	0.436	1.252	0.711	2.203
Brushing teeth with tap water	−0.614	0.442	1.931	1	0.165	0.541	0.228	1.287
Alcohol consumption	0.387	0.410	0.890	1	0.345	1.472	0.659	3.286
Smoking status	0.424	0.357	1.409	1	0.235	1.528	0.759	3.076
Secondhand smoke	0.197	0.282	0.489	1	0.484	1.218	0.700	2.119
Chronic stress (>14 days)	−0.121	0.060	4.054	1	0.044	0.886	0.787	0.997
ST-5 total score	−0.393	0.047	71.007	1	0.000	0.675	0.616	0.740
ST-5 cutoff score	−1.157	0.146	62.785	1	0.000	0.314	0.236	0.418
ST-5 group (moderate to high stress)	2.088	0.290	51.706	1	0.000	8.070	4.568	14.258
Q2	1.203	0.332	13.155	1	0.000	3.330	1.738	6.381
PHQ-9 score	−0.272	0.030	83.583	1	0.000	0.762	0.719	0.808
PHQ-9 (moderate to high depression)	3.064	0.301	103.930	1	0.000	21.404	11.877	38.574

**Table 5:** Multivariable logistic regression analysis identifying independent predictors of MDD.

Factor	B	S.E.	Wald	df	*p*-Value	OR	95% CI	
							Lower	Upper
Congenital disease	1.361	0.338	16.235	1	0.000	3.902	2.012	7.567
High-fat diet consumption	1.501	0.505	8.853	1	0.003	4.487	1.669	12.063
ST-5	1.457	0.332	19.257	1	0.000	4.294	2.240	8.231
PHQ-9 score (0–27)	−0.112	0.052	4.614	1	0.032	0.894	0.807	0.990
PHQ-9 (moderate to high depression)	1.297	0.582	4.976	1	0.026	3.659	1.171	11.439

**Table 6:** Interaction effects of HHV-6 infection and psychosocial, behavioral, and clinical factors associated with MDD risk.

Factor		B	S.E.	Wald	df	*p*-Value	OR	95% CI	
								Lower	Upper
HHV6	by Sex	0.881	0.320	7.574	1	0.006	2.412	1.289	4.517
HHV6	by Faculty	−0.072	0.026	7.584	1	0.006	0.931	0.884	0.979
HHV6	by Science–Arts stream	−0.668	0.220	9.186	1	0.002	0.513	0.333	0.790
HHV6	by Family relationship	−0.252	0.121	4.338	1	0.037	0.777	0.613	0.985
HHV6	by Weight	0.043	0.019	5.393	1	0.020	1.044	1.007	1.083
HHV6	by BMI	−0.125	0.050	6.351	1	0.012	0.882	0.800	0.973
HHV6	by Congenital disease	1.013	0.203	24.986	1	0.000	2.753	1.851	4.096
HHV6	by Fresh fruit consumption	−0.345	0.139	6.108	1	0.013	0.709	0.539	0.931
HHV6	by Vegetable consumption	−0.904	0.332	7.389	1	0.007	0.405	0.211	0.777
HHV6	by High-fat food consumption	0.982	0.359	7.498	1	0.006	2.671	1.322	5.396
HHV6	by stress > 14 days	−0.155	0.045	11.996	1	0.001	0.857	0.785	0.935
HHV6	by ST-5 score (0–15)	−0.206	0.095	4.719	1	0.030	0.814	0.676	0.980
HHV6	by PHQ-9 score (0–27)	−0.178	0.057	9.562	1	0.002	0.837	0.748	0.937
HHV6	by Age and Sex	0.044	0.016	7.227	1	0.007	1.045	1.012	1.080
HHV6	by Science–Arts stream, Faculty, Year of study	−0.019	0.005	12.590	1	0.000	0.981	0.970	0.991
HHV6	by ST-5 score, stress > 14 days, sex	−0.006	0.001	44.219	1	0.000	0.994	0.992	0.996
HHV6	by Congenital disease, exercise > 30 min, sex	0.118	0.049	5.665	1	0.017	1.125	1.021	1.240
HHV6	by Congenital disease, stress > 14 days	0.067	0.026	6.584	1	0.010	1.070	1.016	1.126
HHV6	by secondhand smoke, alcohol consumption, smoking	0.085	0.044	3.765	1	0.052	1.089	0.999	1.186
HHV6	by ST-5, family relationships, stress > 14 days	−0.032	0.005	45.284	1	0.000	0.969	0.960	0.978
HHV6	by PHQ-9 score (0–27), ST-5 score (0–15)	−0.013	0.001	80.850	1	0.000	0.987	0.984	0.990
HHV6	by high-fat food, fermented food, household water use	0.074	0.025	8.480	1	0.004	1.077	1.024	1.132
HHV6	by ST-5 score (0–15), alcohol consumption, smoking	−0.044	0.008	30.685	1	0.000	0.957	0.943	0.972
HHV6	by Q2 by PHQ-9 by ST-5	−0.142	0.018	63.333	1	0.000	0.867	0.837	0.898
HHV6	by BMI, sex	0.044	0.015	8.176	1	0.004	1.045	1.014	1.077

Note: Because multiple interaction tests were performed, Bonferroni correction was applied (α = 0.002). Only interactions remaining significant after correction should be considered exploratory findings requiring independent validation.

## Data Availability

The datasets generated and analyzed during the current study are available from the corresponding author. The authors used ChatGPT version GPT-5.5 (OpenAI, USA) for English language editing and manuscript refinement. All scientific interpretations, analyses, and conclusions were independently reviewed and verified by the authors.

## Supplementary Materials

The following supporting information can be downloaded at: https://www.mdpi.com/article/10.3390/v18060665/s1, Table S1: Baseline characteristics of factors associated with MDD 52 cases and HHV-6 infection.
