# Supplementary material for: Beyond Infection: The Interplay of Salivary Human Herpesvirus 6, Stress, and Host Factors in Major Depressive Disorder
Source: Viruses. 2026 Jun 12;18(6):665. doi: 10.3390/v18060665 (PMC13307731; doi:10.3390/v18060665)
Supplement: Supplementary file 1 [file viruses-18-00665-s001.zip › viruses-4355859-supplementary.pdf]

Supplementary Table S1. Baseline characteristics of factors associated with MDD 52 cases and HHV-6 infection.

Factor	HHV-6			Odds Ratio (95% CI)	p-value
	Positive	Negative	Total		
MDD	30 (57.7%)	22 (42.3%)	52 (100.0%)	NA	NA
Age				NA	0.077
18 years	2 (100.0%)	0 (0.0%)	2 (100.0%)		
19 years	5 (83.3%)	1 (16.7%)	6 (100.0%)		
20 years	14 (63.6%)	8 (36.4%)	22 (100.0%)		
21 years	7 (58.3%)	5 (41.7%)	12 (100.0%)		
22 years	2 (28.6%)	5(71.4%)	7 (100.0%)		
23 years	0 (0.0%)	3 (100.0%)	3 (100.0%)		
Year of study				NA	0.108
First-year	8 (88.9%)	1 (11.1%)	9 (100.0%)		
Second-year	13 (59.1%)	9 (40.9%)	22 (100.0%)		
Third year	5 (41.7%)	7 (58.3%)	12 (100.0%)		
Fourth year	4 (57.1%)	3 (42.9%)	7 (100.0%)		
Fifth year	0 (0.0%)	2 (100.0%)	2 (100.0%)		
Faculty				NA	0.495
Agriculture	3 (100.0%)	0 (0.0%)	3 (100.0%)		
Law	6 (75.0%)	2 (25.0%)	8 (100.0%)		
Business	0 (0.0%)	2 (100.0%)	2 (100.0%)		
Nursing	1 (50.0%)	1(50.0%)	2 (100.0%)		
Pharmacy	4 (66.7%)	2 (33.3%)	6 (100.0%)		
Politics	4 (50.0%)	4 (50.0%)	8 (100.0%)		
Science	1 (50.0%)	1 (50.0%)	2 (100.0%)		
Engineering	3 (50.0%)	3 (50.0%)	6 (100.0%)		
Arts	2 (66.7%)	1 (33.3%)	3 (100.0%)		
Education	0 (0.0%)	2 (100.0%)	2 (100.0%)		
Public Health	6 (60.0%)	4 (40.0%)	10 (100.0%)		
Science and Arts Faculty Group					
Science stream	18 (62.1%)	11 (37.9%)	29 (100.0%)	1.500 (0.494-4.551)	0.473
Arts stream	12 (52.2%)	11 (47.8%)	23 (100.0%)	Ref	
Family relationship				NA	0.094
Very good	11 (50.0%)	11 (50.0%)	22 (100.0%)		
Good	7 (70.0%)	3 (30.0%)	10 (100.0%)		
Normal	12 (70.6%)	5 (29.4%)	17 (100.0%)		
Quarreling	0 (0.0%)	3 (100.0%)	3 (100.0%)		
BMI				NA	0.566
Below normal	6 (50.0%)	6 (50.0%)	12 (100.0%)		
Normal	16 (55.2%)	13 (44.8%)	29 (100.0%)		
Overweight	4 (80.0%)	1 (20.0%)	5 (100.0%)		
Obesity class I	2 (100.0%)	0 (0.0%)	2 (100.0%)		
Obesity class II	2 (50.0%)	2 (50.0%)	4 (100.0%)		
Congenital disease					
Yes	13 (65.0%)	7 (35.0%)	20 (100.0%)	1.639 (0.518-5.184)	0.399
No	17 (53.1%)	15 (46.9%)	32 (100.0%)	Ref	
Family history					
Yes	10 (47.6%)	11 (52.4%)	21 (100.0%)	0.500 (0.162-1.546)	0.226
No	20 (64.5%)	11 (35.5%)	31 (100.0%)	Ref	
Alcohol consumption					
Yes	28 (62.2%)	17 (37.8%)	45 (100.0%)	4.118 (0.718-23.626)	0.094
No	2 (28.6%)	5 (71.4%)	7 (100.0%)	Ref	
Smoking status					0.208

Yes	4 (40.0%)	6 (60.0%)	10 (100.0%)	0.410(0.100-1.681)	
No	26 (61.9%)	16 (38.1%)	42 (100.0%)	Ref	
Secondhand smoke					
Yes	14 (60.9%)	9 (39.1%)	23 (100.0%)	1.264(0.416-3.843)	0.680
No	16 (55.2%)	13 (44.8%)	29 (100.0%)	Ref	
Nutritionally adequate diet					
No	10 (43.5%)	13 (56.5%)	23 (100.0%)	0.346 (0.111-1.082)	0.065
Yes	20 (69.0%)	9 (31.0%)	29 (100.0%)	Ref	
Exercise> 30 min					
No	12 (52.2%)	11 (47.8%)	23 (100.0%)		
1-2 times/week	14 (63.6%)	8 (36.4%)	22 (100.0%)	NA	0.686
3-4 times/week	3 (50.0%)	3 (50.0%)	6 (100.0%)		
5-7 times/week	1 (100.0%)	0 (0.0%)	1 (100.0%)		
Fresh fruit consumption					
No	2 (40.0%)	3 (60.0%)	5 (100.0%)	0.452 (0.069-2.970)	0.400
Yes	28 (59.6%)	19 (40.4%)	47 (100.0%)	Ref	
High-fat food consumption					
No	3 (50.0%)	3 (50.0%)	6 (100.0%)	0.704 (0.128-3.870)	0.685
Yes	27 (58.7%)	19 (41.3%)	46 (100.0%)	Ref	
Fermented food consumption					
No	11 (57.9%)	8 (42.1%)	19 (100.0%)	1.013 (0.323-3.177)	0.982
Yes	19 (57.6%)	14 (42.4%)	33 (100.0%)	Ref	
Household water consumption					
Unclean	11 (61.1%)	7 (38.9%)	18 (100.0%	1.214 (0.387-3.976)	0.717
Clean	19 (55.9%)	15 (44.1%)	34 (100.0%))	Ref	
Boiled or filtered water consumption					
No	13 (65.0%)	7 (35.0%)	20 (100.0%)	1.639(0.518-5.184)	0.399
Yes	17 (53.1%)	15 (46.9%)	32 (100.0%)	Ref	
Brushing teeth with tap water					
Yes	25 (54.3%)	21 (45.7%)	46 (100.0%)	0.238(0.026-2.202)	0.176
No	5 (83.3%)	1 (16.7%)	6 (100.0%)	Ref	
PHQ-9					
No depression	8 (66.7%)	4 (33.3%)	12 (100.0%)		
Mild depression	9 (52.9%)	8 (47.1%)	17 (100.0%)	NA	0.723
Moderate depression	12 (60.0%)	8 (40.0%)	20 (100.0%)		
Severe depression	1 (33.3%)	2 (66.7%)	3 (100.0%)		
ST-5					
No stress	3 (60.0%)	2 (40.0%)	5 (100.0%)		
Mild stress	9 (60.0%)	6 (40.0%)	15 (100.0%)	NA	0.993
Moderate stress	6 (54.5%)	5 (45.5%)	11 (100.0%)		
Severe stress	12 (57.1%)	9 (42.9%)	21 (100.0%)		
